# Are maximum magnitudes of induced earthquakes controlled by pressure diffusion?

**DOI:** 10.1098/rsta.2023.0184

**Published:** 2024-08-09

**Authors:** Cornelius Langenbruch, Mohammad J. A. Moein, Serge A. Shapiro

**Affiliations:** ^1^ Fachbereich Geowissenschaften, Fachrichtung Geophysik, Freie Universität Berlin, Berlin, Germany

**Keywords:** induced earthquakes, seismic hazard, seismology, geo-energy, magnitude, earthquake physics

## Abstract

There is an ongoing discussion about how to forecast the maximum magnitudes of induced earthquakes based on operational parameters, subsurface conditions and physical process understanding. Although the occurrence of damage caused by induced earthquakes is rare, some cases have caused significant economic loss, injuries and even loss of life. We analysed a global compilation of earthquakes induced by hydraulic fracturing, geothermal reservoir stimulation, water disposal, gas storage and reservoir impoundment. Our analysis showed that maximum magnitudes scale with the characteristic length of pressure diffusion in the brittle Earth’s crust. We observed an increase in the nucleation potential of larger-magnitude earthquakes with time and explained it by diffusion-controlled growth of the pressure-perturbed part of faults. Numerical and analytical fault size modelling supported our findings. Finally, we derived magnitude scaling laws to manage induced seismic hazard of upcoming energy projects prior to operation.

This article is part of the theme issue ‘Induced seismicity in coupled subsurface systems’.

## 1. Introduction

Fluid flow in the brittle Earth’s crust is largely maintained by pre-existing faults that are well-oriented for being reactivated in the current tectonic stress field [1,2]. Most induced earthquakes, in particular, the larger ones, occur on these critically stressed faults [3,4]. Injection, production, circulation and storage of fluids change the subsurface pressure and stress conditions and reduce the frictional resistance to sliding. If sufficiently perturbed, faults can slip, and the tectonically accumulated strain energy is released in induced earthquakes [5].

The occurrence of damaging induced earthquakes is rare. However, events like the 1967 Koyna, India, magnitude 6.3, the 2017 Pohang, South Korea, magnitude 5.5, the 2016 Pawnee, USA, magnitude 5.8 and the 2018 Sichuan Basin, China, magnitude 5.2 earthquakes have shown that induced seismicity can cause millions of USD in economic damage, injuries and even loss of life [6–11]. Understanding the nucleation potential of larger-magnitude-induced earthquakes is essential for the sustainable and safe application of energy technologies.

The maximum possible earthquake magnitudes that can be induced by the Enhanced Geothermal System (EGS) stimulation, water disposal, hydraulic fracturing, gas storage and reservoir impoundment are still under discussion [12–18]. Most frequently applied models for injection-induced seismicity relate the maximum magnitude to the net-injected fluid volume [12]. However, events like the M5.4 Pohang earthquake violate the hypothesis that the maximum earthquake magnitude is governed by the volume of injected fluids [19]. Several anomalous hydraulic-fracturing-induced events suggest that the maximum magnitude is ultimately limited by geology rather than operational factors [20,21]. Recently, it has been shown that magnitude scaling with time after the start of fluid injection describes the maximum magnitude data for injection-induced seismicity [22].

In general, pressure diffusion in the pore space and fracture network of rocks has been identified as the dominant physical process triggering injection-induced earthquakes [23–29]. If larger-scale and permeable faults [1,2] exist at a location, pressure will predominantly increase along these faults. We adopt the working hypothesis that induced fault slip occurs along the part of a fault that is destabilized by pressure diffusion. Moreover, if the pressure-perturbed part of a fault is large enough, we assume that a runaway rupture can occur in agreement with Galis *et al*., [14]. However, in this case, we also assume that the rupture length will be in the order of the pressure-perturbed part of a fault. With elapsed time, after the start of energy technology application, the pressure-perturbed part of the fault will grow. If no larger-scale permeable fault exists at a location, we assume that ruptures are limited to the pressure-perturbed rock volume [18]. The maximum possible length scale of fault rupture will be in the order of the pressure perturbation.

Here, we developed a novel physics-based expression for the maximum magnitude of induced earthquakes based on the two conditions outlined above: (i) if pre-existing, critically stressed faults exist at a location, pressure diffusion occurs predominantly along the fault and (ii) the maximum rupture of induced earthquakes is in the order of the pressure-perturbed part of the fault (rock volume) at the occurrence time of an earthquake. According to these assumptions, the nucleation potential of larger-magnitude-induced earthquakes will increase with elapsed time after the start of energy technology application. The later an induced earthquake occurs, the larger it can grow.

Multiple physical mechanisms, including poro-thermo-elastic coupling, and stress changes caused by seismic and aseismic fault slips, have been discussed to contribute to the occurrence of induced seismicity [29–32]. Modelling studies at well-characterized injection locations show that the relative significance of these mechanisms varies from site to site, depending on the physical rock properties, reservoir structure, fault geometry, seismotectonic conditions and distance from injection among others; but, pore pressure diffusion is considered to be the primary mechanism for induced seismicity [28–30,32].

The increase over time of the nucleation potential of larger magnitude events in our model does not give information about the probability of induced earthquake occurrence. It describes the maximum possible magnitude of an earthquake if it occurs. Our model can, for instance, be applied for a physics-based tapering of the Gutenberg–Richter relation and for identifying the maximum magnitudes to be used in seismic hazard and risk modelling prior to operation. The probability rate of event occurrence is largely controlled by the volume of injected fluid as well as the local seismotectonic conditions, and this will decline after the termination of operation [10,27,33].

## 2. Data and methods

The characteristic length scale ( $R_{c}$ ) of pressure diffusion in rocks is well understood [25] and is controlled by hydraulic diffusivity ( D ) and elapsed time ( T ) according to


(2.1) $$R_{c}=\sqrt{4\pi DT}.$$

**Figure 1.**
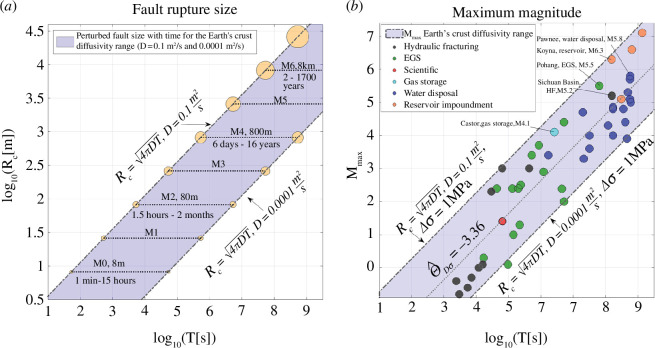
Pressure-diffusion controlled rupture growth and observed maximum magnitudes induced by hydraulic fracturing, EGS, water disposal, gas storage and reservoir impoundment. (*a*) Time (T) of pressure diffusion to reach a distance ( $R_{c}$ ) according to equation (2.1). The blue shaded area shows the growth of the pressure-perturbed part of a fault $\left(R_{c}\right)$ with time according to the diffusivity range of the brittle Earth’s crust ( $D=\;0.1\;m^{2}/s$ to D= 0.0001 ). A fault size (8 m), a potential magnitude *M* = 0 event, is perturbed within 1 min–15 h. A fault of radius 8 km, capable of producing a magnitude *M* = 6 earthquake, is perturbed in 2–1700 years. (*b*) Maximum observed induced earthquake magnitudes $\left(M_{max}\right)$ for case studies of hydraulic fracturing, EGS stimulation, water disposal, gas storage and reservoir impoundment. Each marker corresponds to the maximum magnitude observed for one individual case study. The nucleation time T corresponds to the time from start of fluid injection or reservoir impoundment to occurrence of the maximum magnitude. All maximum magnitudes fall within the pressure-diffusion-controlled range (blue shaded area). The dotted line shows the mean scaling of M_max_ with time ( $\Theta_{D\sigma }=\;-3.36$ , see figure 2). The data shown in B are included in the electronic supplementary material.

No significant increase in pressure, that is, no fault destabilization, is expected for distances larger than $R_{c}$ . If a larger-scale and permeable fault exists at a location, $R_{c}$ can be considered as the pressure-perturbed part of the fault. If no fault exists, $R_{c}$ describes the size of the pressure-perturbed rock volume. In both cases, we assume that the maximum possible rupture is in the order of the characteristic size $R_{c}$ of pressure diffusion. Note that this assumption includes the case where only a significant part of a fault must be pressure perturbed to result in rupture of the complete fault (runaway rupture). We applied diffusivities of $D=10^{-1}\;m^{2}/s$ to $D=10^{-4}$ , the value range reported for the upper brittle Earth’s crust [2], and we present the corresponding growth of $R_{c}$ with time T in figure 1*a*.

**Figure 2.**
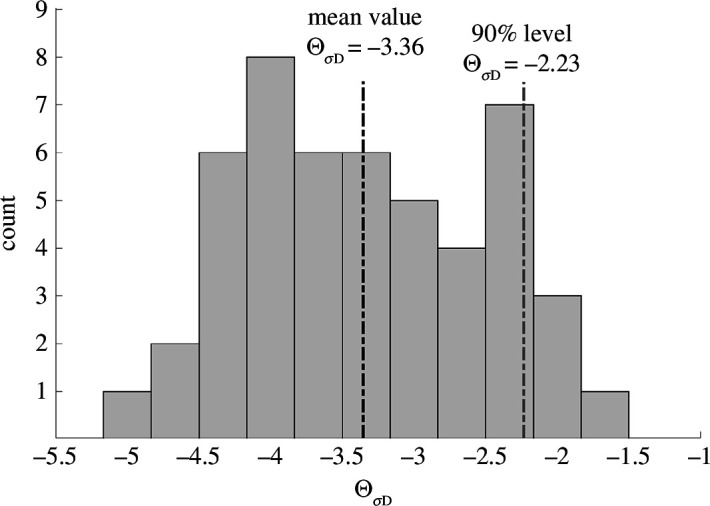
Calculated seismic nucleation constants $\left(\Theta_{D\sigma }\right)$ , expected and upper-bound (90% level) maximum magnitude scaling; $\Theta_{D\sigma }$ calculated for of all case studies is shown in figure 1*b*. The seismic nucleation constants show a value range between $\Theta_{D\sigma }$ = −4.88 and −1.8 with mean value of −3.36 (see also the electronic supplementary material, table S1) ;90% of $\Theta_{D\sigma }$ values fall below $\Theta_{D\sigma }=-2.23$ .

To understand diffusion-based moment magnitude ${(M}_{W})$ growth with time, we considered well-accepted scaling relations between the radius (R) of a circular rupture, seismic moment ${(M}_{0})$ , moment magnitude and stress drop ( Δσ ) [34–37],


(2.2) $$M_{W}=\frac{2}{3}\left({log}_{10}M_{0}-9.1\right),\;\;M_{0}=\frac{16}{7}\;R^{3}\Delta \sigma .$$

If the rupture radius R is of the order of the pressure-perturbed radius ${(R}_{c}),$ the scaling of the maximum magnitude with time is given by


(2.3) $$M_{W}^{max}=\;{log}_{10}T+\Theta_{D\sigma },\;\;\Theta_{D\sigma }={log}_{10}D+\frac{2}{3}{log}_{10}\Delta \sigma -4.7281.$$

Here, we introduced the seismic nucleation constant $\Theta_{D\sigma }$ . It is defined by hydraulic diffusivity and earthquake stress drop. Note that all units in our formulations are expressed in the International System of Units. The seismic nucleation constant corresponds to the magnitude having a nucleation time of 1 s (*T* = 1 s in equation (2.3)). The $\Theta_{D\sigma }$ is a site-specific quantity and controls the characteristic time needed to reach a subsurface state that allows an earthquake of a given magnitude to occur. The nucleation constant $\Theta_{D\sigma }$ effectively considers site-specific tectonic stress conditions as well as the number, orientation and size of pre-existing faults. The $\Theta_{D\sigma }$ will increase with the number and size of critically stressed and pre-existing faults, because the fault and fracture network control the bulk diffusivity considered in equation (2.3). If no larger-scale and permeable fault is present at a location, the diffusivity will correspond to the diffusion process in the pore space of rocks. It suggests that, in addition to operational parameters, subsurface conditions must be considered to understand induced seismic hazards.

Considering a well-accepted average earthquake stress drop of 1 MPa [38] and the Earth’s crust diffusivity range [2], we found that a fault size of 8 m, producing a magnitude *M*_0_ event, is pressure perturbed within 1 min – 15 h. It takes 1.5 h – 2 months to perturb a fault size of 80 m, resulting in a potential M2 earthquake. The characteristic size 800 m, an M4 earthquake, is pressure perturbed within 6 days – 16 years. Finally, a fault of 8 km radius, capable of producing an M6 earthquake, is reached within 2 – 1700 years (see figure 1*a*). The $\Theta_{D\sigma }$ controls the site-specific nucleation time within the range given above. The larger $\Theta_{D\sigma }$ , the shorter is the nucleation time of an earthquake of a given magnitude. Note that equation (2.3) $\left(M_{W}^{max}=\;{log}_{10}T+\Theta_{D\sigma }\right)$ does not explicitly work with the pore-pressure triggering assumption, and it can be understood in a more general way. However, the nucleation constant $\Theta_{D\sigma }$ may be process specific.

## 3. Global compilation of maximum magnitudes of induced earthquakes

To support the derived scaling of the maximum magnitude (equation (2.3)) with time, we gathered a global compilation of induced earthquakes caused by hydraulic fracturing, EGS stimulation, water disposal, gas storage and reservoir impoundment (figure 1*b*). We found that the maximum observed magnitudes of all analysed case studies fall within the diffusion-controlled range (blue shaded area in figure 1*b*).

The damaging M5.5 Pohang earthquake is the most prominent case of violating the maximum magnitude scaling with injection volume [12]. While the injected volume was less than 1/500th of the amount expected to produce an M5.5 event [19], the earthquake fits well into the pressure diffusion–derived time scaling (figure 1*b*). The Pohang earthquake is the largest event related to EGS stimulation, and it had the longest nucleation time (about 2 years) of all EGS stimulation–induced earthquakes to date. It occurred about 2 months after the injection had already been terminated. Pressure diffusion along faults continues even after the injection had been stopped completely [10,26]. Larger-magnitude-induced events, like the Pohang earthquake, occurred on pre-existing faults that were well oriented for being reactivated by fluid injection [19].

The largest earthquake caused by hydraulic fracturing (Mw 5.2) occurred in December 2018 in the Changning shale gas field in the southwest Sichuan Basin, China [11]. Systematic hydraulic fracturing in horizontal wells in the gas field began in 2014, resulting in a dramatic increase in earthquake activity [39]. A nucleation time of 4–5 years fits well into the derived magnitude scaling (figure 1*b*). We used the beginning of systematic hydraulic fracturing as the start time, because detailed information about the injection operations in the field is not available. Local stimulated reservoir volumes for specific boreholes or even stages are also not available. In 2016, the largest earthquake (M5.8) caused by water disposal occurred in Pawnee, Oklahoma [40]. Water injection in the region began about 18 years prior to the Pawnee earthquake, which is in agreement with our diffusion-controlled maximum magnitude model (figure 1*b*).

We also analysed the largest magnitude earthquakes caused by gas injection. Two M4.1 earthquakes occurred about 30 days after the start of gas injection into a depleted oil field at the Castor injection platform offshore Spain in 2013 [41]. Within this time, a fault size of about 1.8 km was pressure perturbed according to equation (2.1) and $D\approx \;0.1\;m^{2}/s$ , which is in agreement with the observed maximum magnitudes (figure 1*b*) and the order of reported diffusivity [41].

In addition to injection-induced seismicity, we analysed the maximum magnitudes of earthquakes likely related to reservoir impoundment [6,7]. To our best knowledge, there is no existing model explaining the maximum magnitudes triggered by reservoir impoundment. Pressure diffusion has also been considered as the physical mechanism triggering these events. The largest reported magnitudes at the Koyna reservoir (India) [6,7], the Polyphyto reservoir (Greece) [42,43] and Lake Hebgen, Montana (USA) [44] are M6.3, M6.6 and M7.1, respectively. Nucleation times of 5, 21 and 44 years after initial filling of the reservoirs fit well into the diffusion-controlled model (figure 1*b*).

## 4. Forecasting maximum induced earthquake magnitudes prior to operation

To develop and calibrate a first-order maximum magnitude model for induced seismic hazard management of upcoming projects, we calculated the seismic nucleation constants (equation (2.3)) of all analysed case studies shown in figure 1*b*. No assumptions about diffusivity or stress drop are needed to calculate $\Theta_{D\sigma }$ . The seismic nucleation constants show a value range between $\Theta_{D\sigma }=-4.88$ and $\Theta_{D\sigma }=-1.80$ and a mean value of ${\hat{\Theta }}_{D\sigma }=-3.36$ (figure 2). As discussed, the seismic nucleation constants correspond to the magnitudes having a nucleation time of 1 s.

Using the mean value of ${\hat{\Theta }}_{D\sigma }=-3.36$ , we computed the expected maximum magnitude scaling with time (table 1). For instance, based on the analysed case studies, maximum earthquake magnitudes characterized by a nucleation time of 2 years show expected values of *M* = 4.1. After 10 years, the expected maximum magnitude increases to *M* = 5.1. We also define an upper-bound scaling of maximum magnitude of induced earthquakes based on the 90% level of calculated seismic nucleation constants $\left(\Theta_{D\sigma }=-2.23\right)$ (see figure 2). Using the upper bound we found maximum magnitudes about 1.1 units higher than the expected ones (see table 1). Expected and upper-bound maximum magnitude scaling with time can be applied for a physics-based tapering of the Gutenberg–Richter relation and for identifying maximum magnitudes to be used in seismic hazard and risk modelling prior to operation. While our model is calibrated using the collected maximum magnitude data, it considers the driving physical process of pressure diffusion to forecast maximum magnitudes.

**Table 1.** Maximum expected and upper-bound maximum magnitude scaling computed according to the mean and upper bound (90% level) of $\Theta_{D\sigma }$ (see figure 2) of case studies analysed in figure 1 *b*.

nucleation time	*M*_max_ upper bound (90%)	*M*_max_ expected
1 h	1.3	0.2
1 day	2.7	1.6
1 week	3.6	2.4
1 month	4.2	3.1
1 year	5.3	4.1
2 years	5.5	4.4
10 years	6.3	5.1
100 years	7.3	6.1

Forecasting single events (*M*_max_) is inherently associated with a large uncertainty. We find that, for a given nucleation time, magnitudes can vary by ±1.5 based on the analysed data (see figure 2). Also, note that our model does not include induced seismicity caused by hydrocarbon extraction [45] and CO_2_ storage [46], because the physical processes triggering seismicity are different in these cases and potentially require process-specific nucleation constants.

## 5. Numerical pressure diffusion modelling

**Figure 3.**
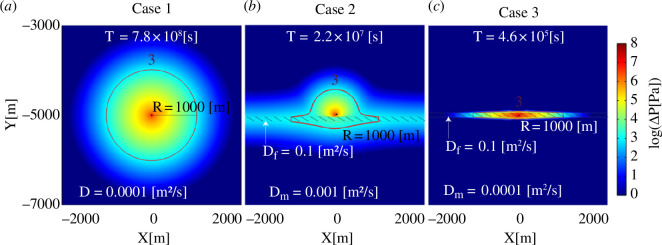
Two-dimensional slices of injection-induced pressure increase caused by fluid injection into the three-dimensional numerical-model scenarios. The figure shows the pressure perturbation at the time corresponding to a perturbed radius of 1000 m (M4.1 earthquake). (*a*) Case 1, a medium with a homogeneous diffusivity and no large-scale pre-existing fault zone. An existing fault and fracture network is effectively included in the model by considering the bulk diffusivity of the medium. (*b*) Case 2, a medium with a pre-existing and permeable fault zone at 50 m distance to the injection point. (*c*) Case 3, a medium with a pre-existing and permeable fault zone that is located directly around the injection point. Depending on the diffusivity structure of the numerical model, the nucleation time of the M4.1 event varies between 5 days and 25 years. The three numerical models (cases 1–3) are characterized by seismic nucleation constants of $\Theta_{D\sigma }=-4.8$ , $\Theta_{D\sigma }=-3.2$ and $\Theta_{D\sigma }=-1.6$ , respectively (see the electronic supplementary material, figure S2 and the electronic supplementary material for details).

To understand how the diffusivity structure and pre-existing faults are related to the nucleation time of an earthquake of a given magnitude, we performed three-dimensional numerical pressure diffusion modelling for three end-member cases: (i) fluid injection into a hydraulically homogeneous three-dimensional rock volume without a larger-scale fault, (ii) injection close to a larger-scale and permeable fault, and (iii) injection directly into a permeable fault (figure 3). Implementation of faults is realized by thin layers of increased diffusivity in the numerical model (see the electronic supplementary material for details).

Based on the observation that induced earthquakes can be triggered by pressure increase just above stress perturbations caused by the Earth’s tides (1–10 kPa), (e.g. [28,47,48]), we defined the pressure-perturbed part of the fault, considering a pressure increase of ≥1000 Pa (figure 3). In all three numerical modelling cases, we present the pressure perturbation at the time the perturbed part of the fault reaches 1000 m (figure 3). A perturbed radius of 1000 m corresponds to about a magnitude 4.1 earthquake (considering ∆*σ* = 1 MPa). Note that in the numerical model case 1, pressure diffusion occurs only through the pore- and fracture-space of the medium because no larger-scale and permeable fault exists. In this case, our model considers that maximum ruptures are of the order of the pressure-perturbed rock volume (refer also to [18]).

In case 1, injection into a low diffusivity medium without a fault, the nucleation time of a magnitude 4.1 earthquake is about 25 years. If fluid injection is taking place close to a fault, the nucleation time reduces to about 250 days. Finally, if fluid injection is taking place directly into a permeable fault, the nucleation time corresponds to only 5 days. In terms of the seismic nucleation constants, the three numerical models correspond to lower-bound ( $\Theta_{D\sigma }=-4.8$ , case 1), intermediate ( $\Theta_{D\sigma }=-3.2$ , case 2) and upper-bound ( $\Theta_{D\sigma }=-1.6$ , case 3) situations, respectively. The diffusivity controlling the seismic nucleation constant (equation (2.3)) is the bulk diffusivity of the medium that is pressure perturbed by the fluid injection. In agreement, the bulk diffusivity controlling the seismic nucleation in numerical model case 2 is between background and fault diffusivity (see also the electronic supplementary material, figure S2).

## 6. Discussion

Recently, the concept of runaway ruptures was introduced by Galis *et al*. [14]. In their model, the authors assume that the pressure perturbation caused by a fluid injection is confined to a reservoir of a characteristic size. Runaway ruptures are defined as ruptures propagating outside of the confined reservoir. Our model is different, because we assume that if a critically stressed fault exists, it is likely permeable and the confinement of the pressure perturbation does not exist. In our model, critically stressed faults are pathways for pressure diffusion and earthquake sources. Our model does not rule out runaway ruptures, because our model assumes that only a significant part of a fault (of the order of the rupture length) must be pressure perturbed to result in the rupture of the complete fault.

However, our assumption of permeable critically stressed faults is supported by observations along boreholes. For instance, the PX-2 well at Pohang, South Korea, crossed the fault that slipped 2 years later after the geothermal reservoir stimulation, and this resulted in the Mw5.5 earthquake. During drilling, more than 160 m^3^ of the drilling fluid was lost into the fault and triggered microseismicity, demonstrating the high permeability and critical stress state of the fault [19]. However, the permeability of faults strongly depends on the fault-zone structure. Often, a low-permeability fault core exists and inhibits fluid flow across the fault. The surrounding damage zone of faults is usually higher [49] and promotes fluid flow along the fault. Damage zones of faults that are optimally oriented for slip in the current stress field are often more permeable than damage zones of faults in less critical orientations because healing and sealing processes have not yet progressed as far. However, we acknowledge that based on experimental studies [50,51] and decades of hydrocarbon exploration [52–54], it is well established that faults exhibit a range of different behaviours.

Our model results in monotonically increasing maximum magnitudes with time. Ultimately, there will be an upper limit of the increase of *M*_max._ This limit is determined by the size of the largest pre-existing fault at a location. Once this fault size is reached, the maximum magnitude will not further increase. In addition, if the pressure and stress perturbations are confined to the reservoir in the sedimentary cover, long-term injection or production of fluid will not result in larger magnitudes. However, all case studies that show larger magnitudes (*M* > 4) correspond to cases where faults in the crystalline basement have been reactivated. Confinement of the pressure and stress perturbation to the sedimentary cover usually does not exist for long-term fluid injections. Interestingly, induced seismicity related to long-term gas production in the Groningen gas field, The Netherlands, is limited to the sedimentary reservoir and the maximum magnitude of M3.6 is smaller than expected [45].

Finally, we present how our model can be used to estimate the induced seismic hazard and risk of upcoming energy technology applications prior to operation. As an example, we discuss the application to the Pohang and Basel EGS case studies. In both cases, a fluid volume of about 12 000 m^3^ was injected into the crystalline basement to stimulate the geothermal reservoir. In the case of the Basel EGS, the injection was planned for a time of about 1 week. The injection at Pohang took place in cycles over a 2-year period. Using the upper bound (90% level) of the seismic nucleation constant ( $\Theta_{D\sigma }=-2.23$ ), we derive a maximum magnitude of *M*_max_ = 3.5 for Basel and *M*_max_ = 5.6 for Pohang (see equation (2.3) and table 1). Note that the estimated magnitudes are close to what has been observed.

The calculated maximum magnitudes can be used for scenario risk modelling [55] prior to operation using Prompt Assessment of Global Earthquakes for Response (PAGER) [56] or other available modelling tools. In the electronic supplementary material, figure S3 shows the result of a PAGER scenario model considering a magnitude 5.5 event at the location of the Pohang EGS site. An M5.5 earthquake at the Pohang EGS site is expected to cause a direct economic loss of $46M and there is a 34% probability of fatalities. This unacceptable risk level could have been identified prior to operation. During the project, the registered seismicity can be used to quantify the evolving risk based on the value at induced risk model as described in [55].

**Figure 4.**
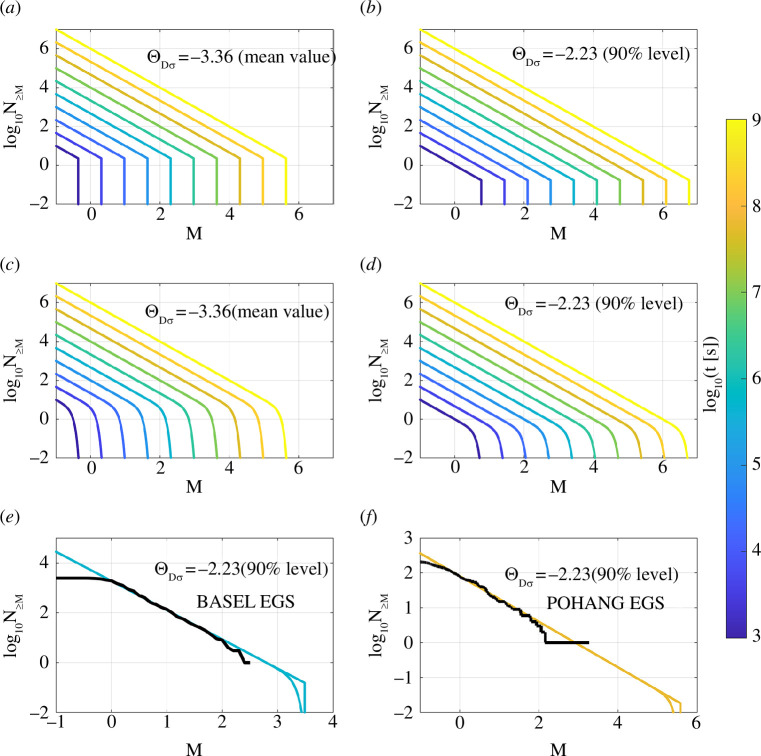
Magnitude distributions with cut-off and tapering according to the derived maximum magnitude scaling with time. (*a*)–(*d*) show synthetic examples. (*a*) Magnitude cut-off using the mean value of ${\hat{\Theta }}_{D\sigma }=-3.36$ . (*b*) Magnitude cut-off using the 90% level of $\Theta_{D\sigma }=-2.23$ . Other parameters used in the simulation are: *b* = 1, constant earthquake productivity of 0.001 events of M≥ 0 per second (*a* = −3 [1/s]). (*c*) Magnitude truncation using equation (2.4) and the mean value of ${\hat{\Theta }}_{D\sigma }=-3.36$ . (*d*) Magnitude truncation using equation (2.4) and the 90% level of $\Theta_{D\sigma }=-2.23$ . Parameters used for the simulation of C and D are: *b* = 1, constant earthquake productivity of 0.001 events of M≥ 0 per second (*a* = −3 [1/s]), *γ* = 5 and *δ* = 10. The colour coding corresponds to the nucleation time used to compute the cut-off and corner magnitudes according to equation (2.3). (*e*) Application of magnitude cut-off and truncation for the Basel EGS case study at the occurrence time of the maximum magnitude. The solid black line shows the reported magnitude distribution at the occurrence time of the maximum magnitude. (*f*) Application of magnitude cut-off and truncation for the Pohang EGS case study at the occurrence time of the maximum magnitude. The solid black line shows the reported magnitude distribution at the occurrence time of the maximum magnitude. Note the different scales of the axes in (*e*) and (*f*).


The classical Gutenberg–Richter law ( ${log}_{10\;}\left(N_{\geq M}\right)=a-bM$ ) has no upper limit of the maximum possible magnitude; *b*-value describs the scaling between small and large magnitudes, and *a* is the earthquake productivity. Some approaches introduce artificial magnitude-tapering functions or magnitude cut-off values in the GR statistics [21,57,58]. In the resulting truncated Gutenberg–Richter law, larger magnitudes are underrepresented and an upper magnitude limit exists. We propose that our derived upper-bound maximum magnitude scaling with time ( $\Theta_{D\sigma }=-2.23$ ) or the mean level of ( ${\hat{\Theta }}_{D\sigma }=-3.36$ ) can be used for a physics-based tapering of the Gutenberg–Richter law for induced seismicity. The derived nucleation constants can be either used as a hard cut-off for the maximum magnitude or as the specific magnitude for tapering the Gutenberg–Richter law (figure 4). For instance, exponential tapering can be applied to the Gutenberg–Richter law according to studies [58–60],

(2.4) $$P\left(M\right)=\beta \left(e^{-\beta \left(M-M_{c}\right)-\gamma e^{\delta \left(M-M_{m}\right)}}\right),M>M_{c}\;,$$

where P(M) is the probability density of the Gutenberg–Richter law, β is related to the *b*-value by β=log(10)b , $M_{c}$ is the magnitude of completeness, $M_{m}$ is a taper parameter that marks the corner magnitude of the distribution and *γ* and *δ* are positive constants [21,58–60]. In the limit of $M_{m}\to \infty$ , the distribution reduces to the untapered Gutenberg–Richter probability density.

In figure 4, we present how the cut-off and tapering (equation (2.4)) approaches can be applied using our maximum magnitude model. Note that the corner magnitude $M_{m}$ and the cut-off magnitude are time-dependent in our model (see equation (2.3)). Depending on earthquake productivity and the choice of $\Theta_{D\sigma }$ , we find that the tapering sometimes only occurs for magnitudes above the maximum statistically expected magnitude (compare with figure 4*b,d*, $\Theta_{D\sigma }=-2.23$ ). It means that the truncation of the magnitude distribution sometimes cannot be identified by a purely statistical analysis of magnitudes in a recorded induced seismicity catalogue. The maximum magnitude of induced earthquake catalogues usually is as large as statistically expected from the Gutenberg–Richter law [13]. However, the tapering above the statistically expected maximum magnitude level is crucial to estimate the occurrence probabilities of larger-magnitude, low-probability earthquakes. Considering low‐probability, high‐impact events is essential, because the risk associated with induced seismicity ultimately depends on whether a large‐magnitude earthquake is triggered [55] (see also [45] for a physically based tapering example).

Figure 4 also shows how our model can be applied at the time of occurrence of the largest magnitudes at Pohang and Basel. Again, we find that the tapering of the magnitude distribution occurs below the observable level of the expected maximum magnitude ( $N_{\geq M}=1$ ). However, the tapering gives important insights into the probability distribution of larger magnitudes. At Pohang, the magnitude cut-off occurs at $M_{w}=5.6$ at the occurrence time of the maximum magnitude, while at Basel, the cut-off is at $M_{w}=3.5$ . In future applications, our model of the maximum magnitude could be combined with the Seismogenic index model [33,61] to describe time- and space-dependent earthquake productivity (the *a*-value) based on operational parameters and the seismotectonic state at an injection location.

## 7. Conclusion

In summary, our analysis demonstrates that the maximum magnitudes of induced earthquake can be explained by pressure diffusion in the brittle Earth’s crust. Because the pressure-perturbed part of a fault is growing with time, the nucleation potential of larger-magnitude-induced earthquakes is increasing. Our model agrees with global observations of hydraulic fracturing, geothermal reservoir stimulation, water disposal, gas storage and reservoir impoundment. Maximum magnitudes of induced earthquakes can be larger than expected from scaling with the injected fluid volume. In some cases, the nucleation time can be more indicative of the physical process controlling the possible maximum magnitude. Characterization of the site-specific hydrogeologic and seismo-tectonic conditions can be useful to understand the nucleation potential of larger induced earthquake magnitudes ( M≥4 ). In particular, the identification of larger-scale, pre-existing and critically stressed faults can help to understand and mitigate induced seismic hazards. The upper-bound maximum magnitude scaling, derived in our study, can be applied for a physics-based tapering of the Gutenberg–Richter relation and to estimate the induced seismic hazard and risk of upcoming energy technology applications prior to operation.

## Data Availability

All data used in this study are included in the supplementary material [62]. References [63–67] are cited in the supplementary information.

## References

[1] Barton CA , Zoback MD , Moos D . 1995 Fluid flow along potentially active faults in crystalline rock. Geology **23** , 683. (10.1130/0091-7613(1995)023<0683:FFAPAF>2.3.CO;2)

[2] Townend J , Zoback MD . 2000 How faulting keeps the crust strong. Geology **28** , 399–402. (10.1130/0091-7613(2000)28<399:HFKTCS>2.0.CO;2)

[3] Walsh FR , Zoback MD . 2016 Probabilistic assessment of potential fault slip related to injection-induced earthquakes: application to north-central Oklahoma, USA. Geology **44** , 991–994. (10.1130/G38275.1)

[4] Schoenball M , Ellsworth WL . 2017 Waveform‐relocated earthquake catalog for Oklahoma and Southern Kansas illuminates the regional fault network. Seismol. Res. Lett. **88** , 1252–1258. (10.1785/0220170083)

[5] National Research Council . 2012 Induced seismicity potential in energy technologies. The National Academies Press.

[6] Talwani P , Acree S . 1985 Pore pressure diffusion and the mechanism of reservoir-induced seismicity. Pure Appl. Geophys. **122** , 947–965. (10.1007/BF00876395)

[7] Gupta HK . 2002 A review of recent studies of triggered earthquakes by artificial water reservoirs with special emphasis on earthquakes in Koyna, India. Earth Sci. Rev. **58** , 279–310. (10.1016/S0012-8252(02)00063-6)

[8] Grigoli F *et al* . 2018 The November 2017 Mw 5.5 Pohang earthquake: a possible case of induced seismicity in South Korea. Science **360** , 1003–1006. (10.1126/science.aat2010) 29700226

[9] Kim KH , *et al* . 2018 Assessing whether the 2017 Mw 5.4 Pohang earthquake in South Korea was an induced event. Science **360** , 1007–1009. (10.1126/science.aat6081) 29700224

[10] Langenbruch C , Zoback MD . 2016 How will induced seismicity in Oklahoma respond to decreased saltwater injection rates? Sci. Adv. **2** , e1601542. (10.1126/sciadv.1601542) 28138533 PMC5262442

[11] Wang S , Jiang G , Lei X , Barbour AJ , Tan X , Xu C , Xu X . 2022 Three Mw ≥ 4.7 earthquakes within the Changning (China) Shale gas field ruptured shallow faults intersecting with hydraulic fracturing wells. J. Geophys. Res. **127** . (10.1029/2021JB022946)

[12] McGarr A . 2014 Maximum magnitude earthquakes induced by fluid injection. J. Geophys. Res. Solid Earth **119** , 1008–1019. (10.1002/2013JB010597)

[13] van der Elst NJ , Page MT , Weiser DA , Goebel THW , Hosseini SM . 2016 Induced earthquake magnitudes are as large as (statistically) expected. J. Geophys. Res. **121** , 4575–4590. (10.1002/2016JB012818)

[14] Galis M , Ampuero JP , Mai PM , Cappa F . 2017 Induced seismicity provides insight into why earthquake ruptures stop. Sci. Adv. **3** , eaap7528. (10.1126/sciadv.aap7528) 29291250 PMC5744472

[15] Afshari Moein MJ , Tormann T , Valley B , Wiemer S . 2018 Maximum magnitude forecast in hydraulic stimulation based on clustering and size distribution of early microseismicity. Geophys. Res. Lett. **45** , 6907–6917. (10.1029/2018GL077609)

[16] De Barros L , Cappa F , Guglielmi Y , Duboeuf L , Grasso JR . 2019 Energy of injection-induced seismicity predicted from in-situ experiments. Sci. Rep. **9** , 4999. (10.1038/s41598-019-41306-x) 30899030 PMC6428893

[17] Li Z , Elsworth D , Wang C , EGS Collab . 2021 Constraining maximum event magnitude during injection-triggered seismicity. Nat. Commun. **12** , 1528. (10.1038/s41467-020-20700-4) 33750772 PMC7943564

[18] Shapiro SA , Krüger OS , Dinske C , Langenbruch C . 2011 Magnitudes of induced earthquakes and geometric scales of fluid-stimulated rock volumes. Geophysics **76** , WC55–WC63. (10.1190/geo2010-0349.1)

[19] Lee KK *et al* . 2019 Managing injection-induced seismic risks. Science **364** , 730–732. (10.1126/science.aax1878) 31123121

[20] Atkinson GM *et al* . 2016 Hydraulic fracturing and seismicity in the western Canada sedimentary basin. Seismol. Res. Lett. **87** , 631–647. (10.1785/0220150263)

[21] Eaton DW , Igonin N . 2018 What controls the maximum magnitude of injection-induced earthquakes? Lead. Edge **37** , 135–140. (10.1190/tle37020135.1)

[22] Shapiro SA , Kim KH , Ree JH . 2021 Magnitude and nucleation time of the 2017 Pohang earthquake point to its predictable artificial triggering. Nat. Commun. **12** , 6397. (10.1038/s41467-021-26679-w) 34737304 PMC8568929

[23] Healy JH , Rubey WW , Griggs DT , Raleigh CB . 1968 The Denver earthquakes. Science **161** , 1301–1310. (10.1126/science.161.3848.1301) 17831340

[24] Zoback MD , Harjes H . 1997 Injection‐induced earthquakes and crustal stress at 9 km depth at the KTB deep drilling site, Germany. J. Geophys. Res. **102** , 18477–18491. (10.1029/96JB02814)

[25] Shapiro SA , Rothert E , Rath V , Rindschwentner J . 2002 Characterization of fluid transport properties of reservoirs using induced microseismicity. Geophysics **67** , 212–220. (10.1190/1.1451597)

[26] Langenbruch C , Shapiro SA . 2010 Decay rate of fluid-induced seismicity after termination of reservoir stimulations. Geophysics **75** , MA53–MA62. (10.1190/1.3506005)

[27] Keranen KM , Weingarten M . 2018 Induced seismicity. Annu. Rev. Earth Planet. Sci. **46** , 149–174. (10.1146/annurev-earth-082517-010054)

[28] Stokes SM , Ge S , Brown MRM , Menezes EA , Sheehan AF , Tiampo KF . 2023 Pore pressure diffusion and onset of induced seismicity. J. Geophys. Res.:Solid Earth **128** , e2022JB026012. (10.1029/2022JB026012)

[29] Moein MJA , Langenbruch C , Schultz R , Grigoli F , Ellsworth WL , Wang R , Rinaldi AP , Shapiro S . 2023 The physical mechanisms of induced earthquakes. Nat. Rev. Earth Environ. **4** , 847–863. (10.1038/s43017-023-00497-8)

[30] Segall P , Lu S . 2015 Injection‐induced seismicity: poroelastic and earthquake nucleation effects. J. Geophys. Res.:Solid Earth **120** , 5082–5103. (10.1002/2015JB012060)

[31] Vilarrasa V , De Simone S , Carrera J , Villaseñor A . 2021 Unraveling the causes of the seismicity Iinduced by underground gas storage at castor, Spain. Geophys. Res. Lett. **48** , e2020GL092038. (10.1029/2020GL092038)

[32] Ge S , Saar MO . 2022 Review: induced seismicity during geoenergy development—a hydromechanical perspective. J. Geophys. Res.: Solid Earth **127** , e2021JB023141. (10.1029/2021JB023141)

[33] Shapiro SA , Dinske C , Langenbruch C , Wenzel F . 2010 Seismogenic index and magnitude probability of earthquakes induced during reservoir fluid stimulations. Lead. Edge **29** , 304–309. (10.1190/1.3353727)

[34] Kanamori H . 1977 The energy release in great earthquakes. J. Geophys. Res. **82** , 2981–2987. (10.1029/JB082i020p02981)

[35] Hanks TC , Kanamori H . 1979 A moment magnitude scale. J. Geophys. Res.: Solid Earth **84** , 2348–2350. (10.1029/JB084iB05p02348)

[36] Madariaga R . 1979 On the relation between seismic moment and stress drop in the presence of stress and strength heterogeneity. J. Geophys. Res.: Solid Earth **84** , 2243–2250. (10.1029/JB084iB05p02243)

[37] Lay T , Wallace TC . 1995 Modern global seismology. Elsevier.

[38] Abercrombie RE . 2021 Resolution and uncertainties in estimates of earthquake stress drop and energy release. Philos. Trans. A. Math. Phys. Eng. Sci. **379** , 20200131. (10.1098/rsta.2020.0131) 33715406

[39] Lei X , Huang D , Su J , Jiang G , Wang X , Wang H , Guo X , Fu H . 2017 Fault reactivation and earthquakes with magnitudes of up to Mw4.7 induced by shale-gas hydraulic fracturing in Sichuan Basin, China. Sci. Rep. **7** , 7971. (10.1038/s41598-017-08557-y) 28801551 PMC5554178

[40] McGarr A , Barbour AJ . 2017 Wastewater disposal and the earthquake sequences during 2016 near Fairview, Pawnee, and Cushing, Oklahoma. Geophys. Res. Lett. **44** , 9330–9336. (10.1002/2017GL075258)

[41] Cesca S , Stich D , Grigoli F , Vuan A , López-Comino JÁ , Niemz P , Blanch E , Dahm T , Ellsworth WL . 2021 Seismicity at the castor gas reservoir driven by pore pressure diffusion and asperities loading. Nat. Commun. **12** , 4783. (10.1038/s41467-021-24949-1) 34376685 PMC8355105

[42] Pavlou K , Kaviris G , Chousianitis K , Drakatos G , Kouskouna V , Makropoulos K . 2013 Seismic hazard assessment in Polyphyto Dam area (NW Greece) and its relation with the" unexpected" earthquake of 13 May 1995 (Ms= 6.5, NW Greece). Nat. Hazards Earth Syst. Sci. **13** , 141–149. (10.5194/nhess-13-141-2013)

[43] Michas G , Pavlou K , Vallianatos F , Drakatos G . 2020 Correlation between seismicity and water level fluctuations in the Polyphyto Dam, North Greece. Pure Appl. Geophys. **177** , 3851–3870. (10.1007/s00024-020-02465-5)

[44] Klose CD . 2013 Mechanical and statistical evidence of the causality of human-made mass shifts on the earth’s upper crust and the occurrence of earthquakes. J. Seismol. **17** , 109–135. (10.1007/s10950-012-9321-8)

[45] Boitz N , Langenbruch C , Shapiro SA . 2024 Production-induced seismicity indicates a low risk of strong earthquakes in the Groningen gas field. Nat. Commun. **15** , 329. (10.1038/s41467-023-44485-4) 38184655 PMC10771524

[46] Glubokovskikh S , Shashkin P , Shapiro S , Gurevich B , Pevzner R . 2023 Multiwell fiber optic sensing reveals effects of CO_2_ flow on triggered seismicity. Seismol. Res. Lett. **94** , 2215–2230. (10.1785/0220230025)

[47] Bachmann CE , Wiemer S , Goertz‐Allmann BP , Woessner J . 2012 Influence of pore‐pressure on the event‐size distribution of induced earthquakes. Geophys. Res. Lett. **39** , 9302. (10.1029/2012GL051480)

[48] Métivier L , de Viron O , Conrad CP , Renault S , Diament M , Patau G . 2009 Evidence of earthquake triggering by the solid earth tides. Earth Planet. Sci. Lett. **278** , 370–375. (10.1016/j.epsl.2008.12.024)

[49] Faulkner DR , Jackson CAL , Lunn RJ , Schlische RW , Shipton ZK , Wibberley CAJ , Withjack MO . 2010 A review of recent developments concerning the structure, mechanics and fluid flow properties of fault zones. J. Struct. Geol. **32** , 1557–1575. (10.1016/j.jsg.2010.06.009)

[50] Ishibashi T , Elsworth D , Fang Y , Riviere J , Madara B , Asanuma H , Watanabe N , Marone C . 2018 Friction‐stability‐permeability evolution of a fracture in granite. Water Resour. Res. **54** , 9901–9918. (10.1029/2018WR022598)

[51] Im K , Elsworth D , Fang Y . 2018 The influence of preslip sealing on the permeability evolution of fractures and faults. Geophys. Res. Lett. **45** , 166–175. (10.1002/2017GL076216)

[52] Smith DA 1966 Theoretical considerations of sealing and non-sealing faults. Am. Assoc. Pet Geol. Bull. **50** , 363–374. (10.1306/5D25B48F-16C1-11D7-8645000102C1865D)

[53] Fulljames JR , Zijerveld LJJ , Franssen R . 1997 Fault seal processes: systematic analysis of fault seals over geological and production time scales (eds P Møller-Pedersen, AG Koestler). vol. **7** . Norwegian Petroleum Society Special Publications. (10.1016/S0928-8937(97)80006-9)

[54] Pei Y , Paton DA , Knipe RJ , Wu K . 2015 A review of fault sealing behaviour and its evaluation in siliciclastic rocks. Earth Sci. Rev. **150** , 121–138. (10.1016/j.earscirev.2015.07.011)

[55] Langenbruch C , Ellsworth WL , Woo J ‐U. , Wald DJ . 2020 Value at induced risk: injection‐Induced seismic risk from low‐probability, high‐impact events. Geophys. Res. Lett. **47** , e2019GL085878. (10.1029/2019GL085878)

[56] Wald DJ , Earle PS , Porter K , Jaiswal K , Allen TI . 2008 Development of the U.S. geological survey’s prompt assessment of global earthquakes for response (PAGER) system. In: Proc. 14th World Conf. Earth. Beijing: IEM.

[57] Muntendam-Bos AG , Grobbe N . 2022 Data-driven spatiotemporal assessment of the event-size distribution of the Groningen extraction-induced seismicity catalogue. Sci. Rep. **12** , 10119. (10.1038/s41598-022-14451-z) 35710738 PMC9203568

[58] Kagan YY . 2010 Earthquake size distribution: power-law with exponent β =1⁄2. Tectonophysics **490** , 103–114. (10.1016/j.tecto.2010.04.034)

[59] Zhuang J , Touati S . 2015 Stochastic simulation of earthquake catalogs, community online resource for statistical seismicity analysis. See http://www.corssa.org.

[60] Vere-Jones D , Robinson R , Yang W . 2001 Remarks on the accelerated moment release model: problems of model formulation, simulation and estimation. Geophys. J. Int. **144** , 517–531. (10.1046/j.1365-246x.2001.01348.x)

[61] Langenbruch C , Weingarten M , Zoback MD . 2018 Physics-based forecasting of man-made earthquake hazards in Oklahoma and Kansas. Nat. Commun. **9** , 3946. (10.1038/s41467-018-06167-4) 30258058 PMC6158231

[62] Langenbruch C , Moein M , Shapiro S . 2024 Data from: Are maximum magnitudes of induced earthquakes controlled by pressure diffusion? Figshare. (10.6084/m9.figshare.c.7249275) PMC11363681 38945164

[63] Bommer JJ , Oates S , Cepeda JM , Lindholm C , Bird J , Torres R , Marroquín G , Rivas J . 2006 Control of hazard due to seismicity induced by a hot fractured rock geothermal project. Eng. Geol. **83** , 287–306. (10.1016/j.enggeo.2005.11.002)

[64] Breede K , Dzebisashvili K , Liu X , Falcone G . 2013 A systematic review of enhanced (or engineered) geothermal systems: past, present and future. Geotherm. Energy **1** , 4. (10.1186/2195-9706-1-4)

[65] Gahalaut K , Rekapalli R . 2022 On the enhanced post-impoundment seismicity in the three gorges reservoir region, China. Nat. Hazards **113** , 1697–1712. (10.1007/s11069-022-05364-1)

[66] Multiphysics C . 2013 Comsol multiphysics reference manual, p. 834, vol. 1084. France: COMSOL.

[67] Yeo IW , Brown MRM , Ge S , Lee KK . 2020 Causal mechanism of injection-induced earthquakes through the Mw 5.5 Pohang earthquake case study. Nat. Commun. **11** , 2614. (10.1038/s41467-020-16408-0) 32457321 PMC7251101

